# Divergence in Glucosinolate Profiles between High- and Low-Elevation Populations of *Arabidopsis halleri* Correspond to Variation in Field Herbivory and Herbivore Behavioral Preferences

**DOI:** 10.3390/ijms20010174

**Published:** 2019-01-05

**Authors:** James Buckley, Foteini G. Pashalidou, Martin C. Fischer, Alex Widmer, Mark C. Mescher, Consuelo M. De Moraes

**Affiliations:** 1 Center for Adaptation to a Changing Environment, Institute of Integrative Biology, ETH Zürich, 8092 Zürich, Switzerland; james.buckley@env.ethz.ch (J.B.); martin.fischer@env.ethz.ch (M.C.F.); 2 Biocommunication Group, Institute of Agricultural Sciences, ETH Zürich, 8092 Zürich, Switzerland; foteini.paschalidou@gmail.com; 3 Plant Ecological Genetics Group, Institute of Integrative Biology, ETH Zürich, 8092 Zürich, Switzerland; alex.widmer@env.ethz.ch; 4 Evolutionary Biology Group, Institute of Integrative Biology, ETH Zürich, 8092 Zürich, Switzerland; mescher@usys.ethz.ch

**Keywords:** preference, performance, defense, glucosinolate, elevation, altitude, herbivore, oviposition, *Pieris brassicae*, *Arabidopsis halleri*

## Abstract

Variation in local herbivore pressure along elevation gradients is predicted to drive variation in plant defense traits. Yet, the extent of intraspecific variation in defense investment along elevation gradients, and its effects on both herbivore preference and performance, remain relatively unexplored. Using populations of *Arabidopsis halleri* (Brassicaceae) occurring at different elevations in the Alps, we tested for associations between elevation, herbivore damage in the field, and constitutive chemical defense traits (glucosinolates) assayed under common-garden conditions. Additionally, we examined the feeding preferences and performance of a specialist herbivore, the butterfly *Pieris brassicae*, on plants from different elevations in the Alps. Although we found no effect of elevation on the overall levels of constitutive glucosinolates in leaves, relative amounts of indole glucosinolates increased significantly with elevation and were negatively correlated with herbivore damage in the field. In oviposition preference assays, *P. brassicae* females laid fewer eggs on plants from high-elevation populations, although larval performance was similar on populations from different elevations. Taken together, these results support the prediction that species distributed along elevation gradients exhibit genetic variation in chemical defenses, which can have consequences for interactions with herbivores in the field.

## 1. Introduction

In light of the widespread and ongoing impacts of human action on natural systems, there is increasing interest in understanding the adaptive responses of plants and other organisms to environmental change [1]. One approach to studying these responses explores genetic and phenotypic variation in plant populations across environmental gradients, including elevation gradients, which can provide a proxy for climatic change as well as associated changes in biotic factors such as herbivory [2,3,4,5]. Rates of herbivory tend to decline with increasing elevation due to decreases in the diversity and abundance of invertebrate herbivores [3,4,5,6]. Given this decline in herbivore pressure, plant defense investment, which entails trade-offs with growth and reproduction [7,8,9] is also generally predicted to decline with increasing elevation. However, two recent reviews of empirical studies failed to identify consistent elevational trends in defense investment [2,3]. Furthermore, while species found at high elevations generally exhibit reduced resistance to herbivores relative to low-elevation species [4,5], there is also some evidence for greater investment in defense traits in high-elevation species [3]. Therefore, the importance of herbivore pressure, relative to other biotic and abiotic factors, in shaping the anti-herbivore defenses of plants living at different elevations, remains unclear.

Challenges in relating changing herbivore pressure to variation in defense investment along environmental gradients arise both because the traits most relevant for defense against herbivores vary across species [3,10,11,12] and as a consequence of the complexity of biotic and abiotic factors that influence defense investment [2,13]. One approach to overcoming the former challenge entails comparing populations of individual species that exhibit broad elevational ranges. Populations of species that are broadly distributed across different elevations can exhibit variation in key functional traits, such as growth rates, physiology and plant size [14,15], similar to that observed among different plant species found at high and low elevations. Furthermore, such patterns are frequently consistent with adaptation to the herbivore pressures and abiotic challenges experienced at different elevations [13,16,17,18,19]. However, studies testing for intraspecific variation in defense traits along elevation gradients have so far been restricted to relatively few taxa [6,13,20,21,22,23,24]. While several studies support the hypothesis that defense investment is reduced in high-elevation populations [20,21,22], others have found that high-elevation populations are more resistant to herbivores [23,24]. Increased investment in defense at high elevations might help to protect leaf tissue that is difficult to produce in these extreme environments, or could be a by-product of an increased general stress response to harsh environmental conditions [2,3].

Plants produce defensive secondary metabolites both constitutively and in response to herbivore attack, with the relative dependence on constitutive and herbivore-induced defenses being thought to depend on the predictability of attack in natural populations [25]. Comparing species found at different elevations has revealed greater defense inducibility in low-elevation species [26]; however, consistent with the above prediction, direct measurements of chemical defenses in populations of a single species have revealed greater inducibility of chemical defenses at high elevations where herbivory is more sporadic [6,21]. On the other hand, constitutive levels of chemical defenses can show both increasing and decreasing trends with elevation depending on the compounds assayed [3,22,27,28]. Surprisingly, relatively few studies found clear associations between intraspecific levels of particular chemical defenses and rates of herbivory along elevation gradients, perhaps reflecting the complexity of abiotic and biotic factors that shape the evolution of plant defense and stress responses [2,3] or confounding environmental effects that arise when plant defenses are measured for plant tissues collected in the field. Performance assays conducted under controlled environmental conditions in order to avoid such environmental effects have revealed that high-elevation plant populations can be more palatable than low-elevation populations [6,20,21], yet in field experiments other studies have shown high-elevation populations to be less palatable to herbivores [23,29].

Although herbivore performance assays are often employed to test for differences in defense investment along elevation gradients, only a few studies have assessed variation in herbivore preference for plant genotypes from different elevations [30,31]. Furthermore, none of these studies assayed both herbivore preference and offspring performance, despite both being important for understanding the effects of variation in defense investment on interactions with herbivores. Ovipositing insects are generally predicted to select plants that are best for the development of their offspring (the “preference-performance” or “mother knows best” hypothesis) [32]. Under this hypothesis ovipositing insects might be predicted to select the least-well defended plants e.g., [33]. However, the inconsistent support for this prediction in the literature suggests that herbivore preference is influenced by many different and interacting factors [32]. For example, oviposition preference can be associated with variation in mechanical defenses, such as leaf trichome production [34,35], but also traits not obviously linked to defense, such as the age of the plant [36] and plant size [35,37]. Furthermore, particular chemical defense traits can be either attractive or repellent depending on the level of ecological specialization of the ovipositing insect [33]. Nevertheless, despite the potential complexity of explanatory factors, the weight of evidence suggests that variation in chemical defense cues can be important in shaping female oviposition behavior on different plant genotypes e.g., [38,39]. More broadly, intraspecific variation in chemical defenses is thought to be an important mechanism driving the assembly of herbivore communities [40,41]; yet few studies attempt to correlate changing investment in plant defenses within species along elevation gradients and plant interactions with their local herbivore communities (notable exceptions include [20,21]).

Plants in the family Brassicaceae are particularly well-suited for testing the effects of intraspecific defense variation on plant-herbivore interactions, as they have a well-characterized chemical defense system involving the production of glucosinolates, defensive compounds that break down into a range of compounds toxic for many herbivores [42]. Glucosinolates show high diversity within and among species, both in absolute quantities produced and in relative proportions of the many known structurally different glucosinolates [43,44,45]. Two such groups of particular interest for the present paper are the methionine-derived glucosinolates (a subclass of aliphatic glucosinolates) and the tryptophan-derived glucosinolates (also known as “indole glucosinolates”). In the rest of this paper, we will use the terms aliphatic glucosinolates and indole glucosinolates for these groups. Typically, the feeding of generalist herbivores is negatively affected by higher total glucosinolate levels in leaves [46,47]; consequently, high rates of herbivory by generalist species are predicted to select for increased investment in total glucosinolates [48]. However, many herbivores that specialize on Brassicaceae can detoxify glucosinolates, avoiding their toxic breakdown products [49], and specialists sometimes use the presence of glucosinolates as a stimulatory factor for feeding and oviposition [50,51]. Indeed, increasing total glucosinolate concentrations are associated with higher specialist herbivore abundance in the field [47,52], suggesting that plants may face selection for reduced glucosinolate concentrations to avoid detection by specialists. Alternatively, even greater investment in glucosinolates might be necessary to challenge the specialist’s detoxification mechanisms [48], although specialists have been observed to tolerate the high levels of glucosinolates present in flowers [53]. Another strategy to promote resistance to both specialist and generalist herbivores is to invest in a greater diversity of glucosinolates (altering glucosinolate profiles), through either quantitative or qualitative changes in the amounts of individual glucosinolates.

Genetic variation in glucosinolate profiles is associated with different herbivore communities on plants in the field [40,41], and can affect rates of herbivore damage in common garden field experiments [52]. Such variation has also been linked to variation in preference of specialist herbivores for different genotypes of *Arabidopsis thaliana* [54] and *Brassica oleracea* [55]. In particular, the indole glucosinolate class has been identified as an important oviposition stimulant for specialist butterflies, including *Pieris* sp. [50,56] and *Plutella xylostella* [51]. Despite these observed effects on preference, several studies have found that variation in glucosinolate profiles has limited effects on the performance of *Pieris* and other specialist larvae [38,46,57]. Increasing relative investment in different biosynthetic groups of glucosinolates, for example tryptophan-derived indole glucosinolates or methionine-derived aliphatic glucosinolates, could therefore alter the interactions of a host plant with its herbivore community, although the outcome may depend on the ecological specialization of the herbivore and might differ for herbivore preference and offspring performance. It is therefore important to consider both herbivore preference and performance when examining the effects of chemical defense variation on herbivores.

The current study explores elevational variation in the chemical defense phenotype and its role in herbivore resistance of *Arabidopsis halleri*, a Brassicaceous perennial herb which is strictly outcrossing but highly clonal and tends to be found in human-disturbed alpine meadows from around 300 to 2400 m above sea level in the Alps. Because this species exhibits a fragmented distribution across a broad elevational range, it provides an attractive study system for exploring the effects of genetic variation in glucosinolate defenses for herbivores. *A. halleri* is known to produce at least 10 different glucosinolates in the leaves [43], of which several are induced by herbivory and may therefore be involved in defense against herbivores [58]. Furthermore, previous research has revealed genomic signatures of selection associated with both abiotic and biotic factors in this species [59,60].

We sampled wild populations of *A. halleri* to explore whether rates of herbivory in the field were associated with elevation and constitutive levels of glucosinolates assayed using greenhouse-grown plants from the different populations. We then conducted preference assays to test whether these specialist butterflies can distinguish between populations from different elevations and whether their preferences correspond to the performance of larvae on plants from these different populations. Specifically, we asked the following questions: (i) How do rates of herbivory in the field change with increasing elevation? (ii) Is variation in constitutive glucosinolate composition in the greenhouse related to elevation and levels of field herbivory? (iii) Do specialist herbivores show variation in egg-laying preference for populations from different elevations, and is female preference consistent with differences in larval performance? Together, our results provide insight into the extent of within-species variation in chemical defenses along elevation gradients, but also contributes to the increasing volume of literature linking variation in constitutive defense investment along environmental gradients with herbivore damage in the field.

## 2. Results

### 2.1. Rates of Herbivore Damage in the Field Decline with Elevation and Are Associated with Increasing Investment in Indole Glucosinolate Production

**Figure 1:**
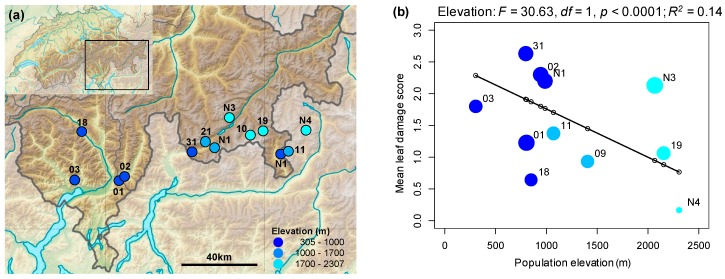

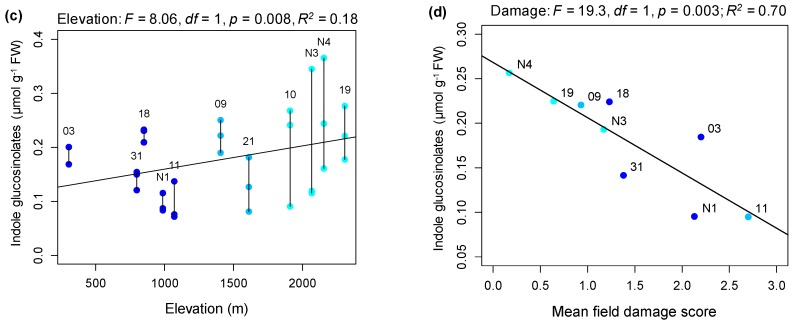
Variation in damage to plants in the field and associations with constitutive leaf chemical defenses. (**a**) Distribution of all 13 study populations of *Arabidopsis halleri* with different colors indicating the broad elevation class of a population. (**b**) Regression of average damage score (based on percentage of leaves damaged) per population with point size weighted by number of patches surveyed, and a line joining fitted value estimates from the corresponding linear model. (**c**) Regression of total indole glucosinolates (in micromoles per gram of fresh tissue, µmol g^−1^ FW) against elevation (with samples from the same population joined by black vertical lines). (**d**) Regression of mean total indole glucosinolates per population against mean field damage score. The significance of the effect of elevation and adjusted R-squared is given for (**b**–**d**). Points in regression plots are labelled by population identifiers.

**Figure 2:**
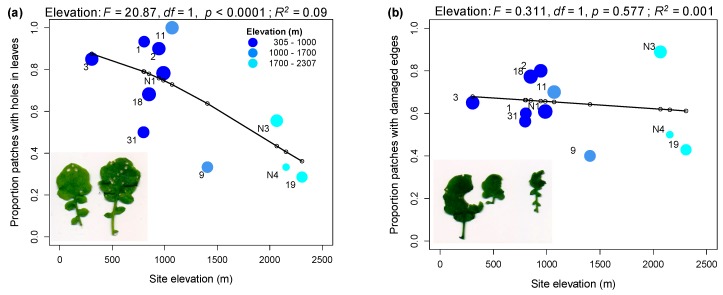
Variation in the proportion of plant patches in the field showing different damage types. (**a**) proportion of patches with holes in leaves. (**b**) proportion of patches with damaged leaf edges. Point size is scaled by sample size (number of patches) and a line joins fitted value estimates from the corresponding linear model with elevation as a factor. Points are labelled by population identifiers.

Early season (2016) field surveys of twelve *A. halleri* populations at elevations between 305 and 2307 m a.s.l (above sea level) (Table S1; Figure 1a) showed a decline in mean leaf damage score with increasing elevation (*R*^2^ = 0.14, *p* < 0.0001; Figure 1b). However, while the proportion of plants with leaf holes declined with increasing elevation (*R*^2^ = 0.09, *p* < 0.0001; Figure 2a), the proportion of patches with damaged edges showed no trend with elevation (*R*^2^ = 0.001; *p* = 0.577; Figure 2b). The herbivores associated with the different types of damage were difficult to observe in the field, and collections made with sticky traps did not identify a dominant herbivore in the populations surveyed; however, the nature of the damage suggested that beetles, small Lepidopteran larvae and molluscs were responsible. Eggs, which were likely laid by the Brassicaceae specialist *Pieris napi*, were found on plants only at two low-elevation populations (Aha03 and Aha18; JB personal observation).

Plants collected from the field and grown in a controlled greenhouse environment for six years (since 2012, with the exception of Aha31 and Aha03 samples that had been in the greenhouse for just over one year; Table S1) were used to assay chemical defense investment, specifically production of glucosinolates. We observed only the methionine-derived “aliphatic” glucosinolates and tryptophan-derived “indole” glucosinolates. Across eleven populations, we identified seven aliphatic glucosinolates (accounting for 97.7% of the total average glucosinolate content) and two indole glucosinolates (2.3% of the total; Table S2). Within populations, the proportion of indole glucosinolates varied 2.5-fold, from 1.3% of total glucosinolates in the low-elevation population AhaN1 to 3.3% for the high-elevation population Aha19 (Table S3). Controlling for variation in plant size at the time of sampling, variation in total aliphatic glucosinolates was not significantly explained by elevation (*p* = 0.461; Figure S1), whereas total indole glucosinolates showed a significant positive association with elevation (*p* = 0.008, *R*^2^ = 0.181; Figure 1c). There was also a positive, though non-significant relationship between mean total aliphatic and mean total indole glucosinolates across populations (*p* = 0.083, Figure S2a). After removing two particularly large plants from the recently sampled population Aha03, plant size (rosette width) did not significantly explain either variation in total aliphatic glucosinolates (*F*_1,28_ = 2.49, *p* = 0.126) or variation in indole glucosinolates (*F*_1,28_ = 1.59, *p* = 0.217). Yet, the significant positive relationship between elevation and indole glucosinolates remained (*F*_1,28_ = 5.51, *p* = 0.026, *R*^2^ = 0.14). 

Increasing mean damage scores for field populations were associated with declining mean indole glucosinolates per population (*F*_1,7_ = 19.31, *p* = 0.003; *R*^2^ = 0.70; Figure 1d), but this trend was not observed for aliphatic glucosinolates (*p* = 0.109, Figure S2b). When field damage was partitioned by feeding type, variation in indole glucosinolates was not significantly associated with the proportion of patches with damaged leaf edges (*p* = 0.492; Figure S2c), but was negatively associated with the proportion patches with hole damage (*R*^2^ = 0.46; *p* = 0.026; Figure S2d).

### 2.2. A Specialist Herbivore (Pieris brassicae) Prefers Plants from Low-Elevation Populations, despite No Associated Enhancement of Offspring Performance

To determine whether herbivore preference and performance reflected the observed patterns of field damage and constitutive glucosinolate production across populations, we conducted two experiments using the specialist herbivore *Pieris brassicae* and *A. halleri* plants cloned from plants collected from the field and then grown under greenhouse conditions for at least one year.

#### 2.2.1. Preference Experiment 1: Assessing Population-Level Effects on Preference

**Figure 3:**
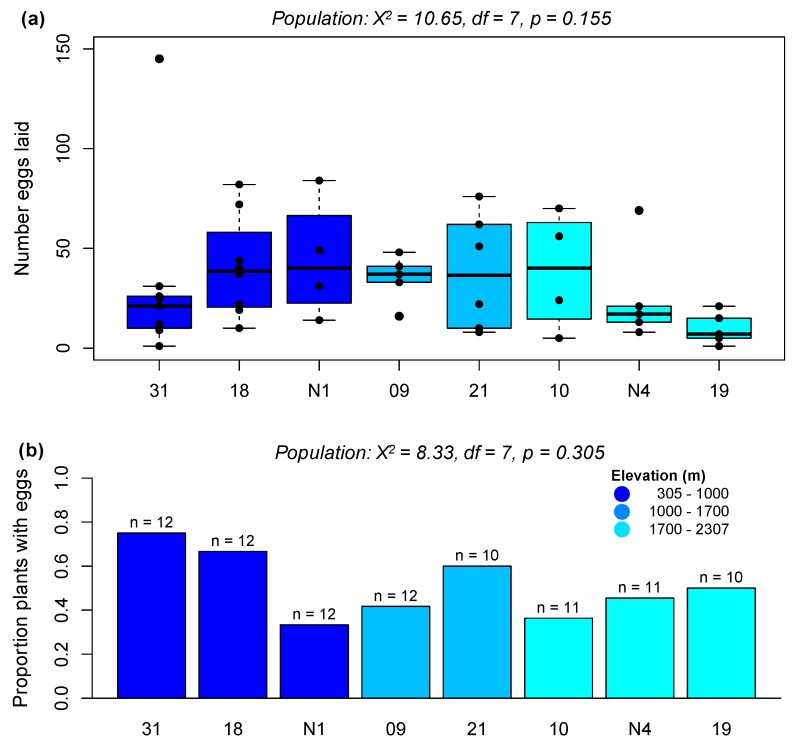
Preference of female *Pieris brassicae* for *A. halleri* populations from different elevations. (**a**) Boxplot showing the number of eggs laid per plant over a 20 h period (excluding all plants that received no eggs). (**b**) Proportion of plants on which eggs were laid in a 20 h period. The number of plants tested in (**b**) is given above each bar. Statistics are given for the effect of population in a negative binomial model, for (**a**), or using a binomial model for (**b**), after accounting for variation among experimental rounds.

To test for variation in *P. brassicae* preference for different populations of *A. halleri*, we conducted randomized choice assays over a 20 h-period using two female *P. brassicae* butterflies and four randomly selected plants per experimental cage. There were eight cages (32 plants) in each of three experimental rounds (91 plants in total)—although one plant, from an intermediate-elevation population (Aha21), on which 417 eggs were laid was excluded from our analyses. Plant population did not significantly explain variance in the number of eggs laid at 20 h (*χ*^2^ = 10.4, *df* = 7, *p* = 0.168), and the effect of population on number of eggs laid remained non-significant even when excluding all plants on which females did not lay eggs (*χ*^2^ = 10.65, *df* = 7, *p* = 0.155; Figure 3a). The number of eggs laid per plant was also not significantly associated with rosette width (*χ*^2^ = 0.63, *df* = 1, *p* = 0.429), plant height (*χ*^2^ = 0.17, *df* = 1, *p* = 0.685), or plant health (*χ*^2^ = 0.59, *df* = 1, *p* = 0.444); however, there was a non-significant effect of experimental round on number of eggs laid (*χ*^2^ = 4.76, *df* = 2, *p* = 0.09). The proportion of plants on which eggs were laid at 20 h also did not significantly vary among populations (*χ*^2^ = 8.33, *df* = 7, *p* = 0.305; Figure 3b), but did vary among the three experimental rounds (*χ*^2^ = 6.53, *df* = 2, *p* = 0.04): butterflies laid eggs on 34.4% plants in round one, 58.1% plants in round two, and 63.0% of plants in round three. Despite the absence of significant population effects, we observed egg-laying behavior consistent with reduced preference for high-elevation populations: specifically, the two lowest-elevation *A. halleri* populations used in this experiment showed the highest mean number of eggs per plant (Aha31 = 23.3 eggs, Aha18 = 27.2 eggs), while the two highest-elevation populations showed the lowest number of eggs (AhaN4 = 11.6, Aha19 = 4.9). We therefore repeated this experiment at a single time point to reduce variance associated with experimental rounds and focused on just these four populations.

#### 2.2.2. Preference Experiment 2: Assessing Elevation Effects on *P. brassicae* Preference

To test for an effect of elevation on preference, we used plants from the two lowest elevation sites (Aha31, Aha18, <850 m above sea level, a.s.l.) and the two highest elevation sites (AhaN4, Aha19; >2155 m a.s.l.). As noted, these sites showed the greatest differences in mean number of eggs laid in preference experiment 1 (summarized in Table S4). Focusing on fewer populations also made if feasible to test whether larval performance varied on low and high-elevation populations consistent with female preference. Given that we were interested in the effect of elevation, the populations were pooled into ‘low’ and ‘high’ elevation classes for statistical analysis.

**Figure 4:**
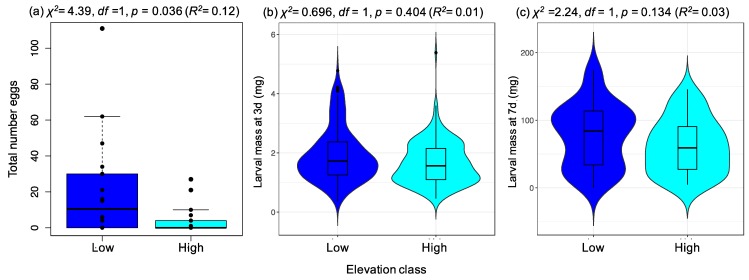
Association between *P. brassicae* preference and larval performance for *Arabidopsis halleri* plants from low and high-elevations. (**a**) Boxplot showing variation in female preference (number of eggs laid after 20 h) for the different elevation classes (with individual data points given), and violin plots showing variation in individual larval mass among elevation classes after (**b**) 3 and (**c**) 7 days of feeding. As a result of the large number of data points in (**b**,**c**), violin plots are presented to indicate the density of observations for particular larval mass values. Within each probability density graph is a boxplot with the dark horizontal line representing the median mass value for that elevation class. For each plot, the significance of the effect of elevation (*x*-axis) on the response variable is given using either a negative binomial model (**a**), or linear mixed effects models (**b**,**c**). Elevation class is represented by two high- and two low-elevation populations.

We found a significant effect of elevation class on the number of eggs laid (*p* = 0.036, *R*^2^ = 0.12; Figure 4a), with on average 5.1× more eggs laid on plants from low-elevation than high-elevation sites (low-elevation mean = 20.4 eggs per plant +/− S.E 6.9; high-elevation mean = 4.0 eggs per plant +/− S.E. 1.9). Interestingly, twice as much variance in egg-laying rate was explained by plant population (not grouped by elevation) than by elevation class (population effect: *R*^2^ = 0.24; *χ*^2^ = 9.20, *df* = 3, *p* = 0.027), suggesting that population-level differences might be more important for shaping egg laying preference than elevation alone. The proportion of plants on which eggs were laid did not significantly vary with respect to elevation (*χ*^2^ = 1.01, *df* = 1, *p* = 0.315) or with average rosette area (*χ*^2^ = 1.71, *df* = 1, *p* = 0.190). Furthermore, plant populations from different elevations did not vary in rosette area (Kruskal–Wallis test: *p* = 0.217) or health score (Kruskal–Wallis test: *p* = 0.500), and neither factor significantly explained variation in the number of eggs laid (*LR-stat =* 3.97, *df* = 2, *p* = 0.138). The mean numbers of eggs laid on the four populations were similar to those observed in preference experiment 1 (Table S4).

Although we observed reduced preference for the high-elevation populations, larval mass did not significantly differ on plants from low- and high-elevation populations after 3 days (log transformed response: *χ*^2^ = 0.70 *df* = 1, *p* = 0.404; Figure 4b) or 7 days (log-transformed response: *χ*^2^ = 2.24, *df* = 1, *p* = 0.134; Figure 4c). Across both time points, the proportion of variance in larval mass explained by elevation was consistently low (3 days: *R*^2^ = 0.01, 7 days: *R*^2^ = 0.03), and instead a greater proportion of variance was explained by individual plant effects (3 days: full mixed model *R*^2^ = 0.62, 7 days: full mixed model *R*^2^ = 0.27), suggesting that larvae on a plant developed at similar rates.

## 3. Discussion

Our data show that rates of herbivory on field populations of *Arabidopsis halleri* decline with increasing elevation and that this variation in field damage is associated with an elevational cline in constitutive glucosinolate profiles. Relative to low-elevation populations, plants from high-elevation populations produced a greater proportion of indole glucosinolates when grown under common environmental conditions and also suffered lower levels of herbivore damage in the field. Furthermore, experimental assays revealed reduced preference of a specialist herbivore for plants from high-elevation populations, despite similar larval performance on plants from the two highest and two lowest elevation populations. To our knowledge, this is one of only a few studies to show variation in herbivore preference for plants originating from different elevations. Furthermore, although our results are correlative in nature, these findings support the hypothesis that genetic variation in plant defenses along elevation gradients is associated with variation in herbivore pressure among natural populations.

### 3.1. Rates of Herbivore Damage in the Field Decline with Elevation and Are Associated with Increasing Investment in Indole Glucosinolate Production

Consistent with previous studies, we found that total levels of herbivore damage in the field declined with increasing elevation [2,3,61]. However, this trend was only observed for one type of damage, leaf holes, characteristic of feeding by beetles and young Lepidopteran larvae. The absence of a decline in leaf-edge damage with elevation could suggest that the maximum elevation at which *A. halleri* is found in our study (Aha19 at ~2300 m above sea level) is still suitable for many invertebrate herbivores. Supporting this, field surveys on natural populations of a related species, *Arabis alpina*, showed that levels of leaf edge (or chewing) damage were most strongly reduced in populations found higher than 2300 m [6]. More focused sampling of the herbivore community would be necessary to reveal the major herbivores attacking *A. halleri* in these field populations.

Decreasing rates of herbivore damage with increasing elevation could either drive selection for reduced investment in plant chemical defenses at high elevations or arise as a consequence of plants at high elevations being better defended. A recent review found conflicting evidence for these two explanations [3], with certain groups of chemical defenses (including secondary defensive metabolites) showing no association with elevation, while others (flavonoids) increasing with elevation. In the current study, we found that investment in total glucosinolates did not significantly vary with elevation, but that the relative proportion of indole glucosinolates significantly increased with elevation, suggesting that high-elevation populations invest more in indole glucosinolate production than low-elevation populations. Interestingly, this shift in glucosinolate profiles was significantly and negatively correlated with leaf damage in the field, which might explain the reduced levels of herbivore damage observed in high-elevation populations. The effect of indole glucosinolates relative to aliphatic glucosinolates on herbivores is complex and tends to differ between generalist and specialist herbivores e.g., [46]. However, indole glucosinolates, rather than total or aliphatic glucosinolates, have been associated with defense against parasitic plants [62], resistance to infection by the pathogen *Phytophthora infestans* [63] and reduced performance of the specialist herbivores *Pieris brassicae* and *Athalia rosae* on different wild populations of *Brassica oleracea* [64,65]. Enzyme hydrolysis of indole glucosinolates following tissue disruption yields breakdown products distinct from those of aliphatic glucosinolates [42,66], which may partly explain the differing biological effects of these glucosinolate classes. Together, these lines of evidence suggest that an increasing proportion of indole glucosinolates in leaves could be responsible for reduced rates of damage from generalist and specialist field herbivores at high elevations. However, given that our results are based only on significant correlations, additional field experiments, potentially using synthetic compounds or plant genotypes differing in indole glucosinolate production, would be necessary to explore the causal nature of this relationship. It should also be noted that other features of the abiotic environment that vary along elevation gradients could simultaneously drive both the change in indole expression and rates of herbivore damage, without a causal link between herbivory and defense investment [3]. 

The observed shift in glucosinolate profiles would also be consistent with plants at low elevations investing less in indole glucosinolates to avoid oviposition by *Pieris* butterflies, which are attracted by the presence of indole glucosinolates [50,56]. However, this explanation is not consistent with our observation for the lower number of eggs laid on high-elevation populations relative to low-elevation populations. An alternative explanation for the increasing investment in indole glucosinolates with elevation is that increasing abiotic stress at high elevations could alter broad physiological stress responses, with associated impacts on the relative production of indole glucosinolates. Total glucosinolate investment has been observed to covary with abiotic factors such as aridity [67] and nutrient status [68]. For example, increasing levels of potassium in local soils, has been associated with increasing relative indole investment across species [68]. Variation in soil types and geology across the Alps could affect the nutritional status of soils in different areas and therefore potentially plant defense investment. However, many abiotic factors vary independently of elevation [69], which makes testing the role of different abiotic stressors in shaping defense variation challenging.

The absence of a change in total constitutive glucosinolates with elevation contrasts with the increased investment in total leaf glucosinolates previously reported for *Cardamine* species found at high elevations (relative to low-elevation *Cardamine* sp.) [26]. However, that study sampled leaves directly from the field, making it difficult to determine whether the observed differences in defensive chemistry are genetically-based, driven by the environment, or both. Our results, obtained from plants grown in the greenhouse for multiple years, support the hypothesis that these populations have genetically diverged in their relative investment in indole and aliphatic glucosinolates, and hence that this difference is likely adaptive. Evidence for genetically-based clines in chemical defense traits has also been observed along broad latitudinal gradients [70], suggesting that genetic variation in plant chemical defense investment is likely widespread within species distributed across spatial environmental gradients. 

### 3.2. A Specialist Herbivore Prefers Plants from Low-Elevation Populations despite No Associated Enhancement of Offspring Performance

Two separate preference experiments using the specialist herbivore *Pieris brassicae* revealed reduced egg-laying rates on plants from the highest elevation populations, yet we found no corresponding change in larval performance. Variation in preference was not explained by differences among the plant populations in plant size or health, but could reflect underlying differences among the populations in physiological status or in chemical and morphological traits linked to plant defense. Given the changing investment in indole glucosinolates with elevation described above, this raises the interesting hypothesis that reduced preference for high-elevation populations may be driven by shifts in population glucosinolate profiles. Although we were unable to directly measure glucosinolate levels in plants used in preference experiments as a consequence of larvae consuming almost all the leaf material available by day 7, both preference experiments used plants cloned from the parental material sampled for the constitutive glucosinolate component of this study, so plants in both experiments would be predicted to show similar glucosinolate profiles.

The reduced egg laying preference for high-elevation populations is consistent with the exposure of high-elevation populations to fewer herbivores in the field and their greater proportional investment in indole glucosinolates (Table S4). By contrast, total glucosinolate investment varied among populations independent of elevation (Table S4), and therefore was not consistent with differences in the mean number of eggs laid per plant. The direction of this correlation between indole glucosinolates and number of eggs laid, might suggest that increasing proportions of indole glucosinolates repels *P. brassicae* oviposition. Yet such a hypothesis is contrary to experimental studies on other Brassicaceae species in which increasing indole glucosinolate concentration stimulates *Pieris* oviposition. For example, *Arabidopsis* mutants with low indole glucosinolate production received significantly fewer *Pieris rapae* eggs in preference assays [56], and *Pieris brassicae* oviposition was also stimulated by increasing levels of the indole glucosinolate glucobrassicin in *Brassica oleraceae* [50]. However, it should be noted that increasing amounts of the indole glucosinolate breakdown product indole-3-acetonitrile reduced egg laying rates in the same study [56]. Our data showed low levels of glucobrassicin in *A. halleri* relative to other glucosinolates (on average just 0.2% of the glucosinolate mix). The effect of the more abundant indole glucosinolate in *A. halleri*, which we provisionally identified as 4-methoxyglucobrassicin (on average 2.1% of the glucosinolate mix), on *P. brassicae* oviposition preference is not clear, although it is known to promote oviposition in another specialist herbivore, *Plutella xylostella* [51]. Testing whether oviposition of *P. brassicae* is stimulated or deterred by the abundance of these indole glucosinolates might provide insight into why indole glucosinolate production varies with elevation. As the correlative evidence presented here cannot elucidate the causal nature of the relationships between changing glucosinolate profiles and herbivore preference, future experiments using synthetic indole glucosinolates would help to determine whether these different indole glucosinolates play an important role in *P. brassicae* oviposition preference. Indeed, at least one previous report of correlations between glucosinolate profiles and generalist herbivore performance [38] identified individual glucosinolates that could potentially explain the observed effects, but which were later found, through further experimental work, not to account for the observed differences in herbivore performance [46].

Alternative explanations for our observed associations are also possible, as physiological or morphological traits that were not measured in our study could drive the reduced preference for high-elevation populations. Plant size, for example, could affect the ‘apparency’ of plants in choice assays, perhaps resulting in reduced preference for or damage to smaller plants independent of defensive chemistry [37,54]. On the other hand, *Pieris brassicae* has been observed to prefer younger, and thus smaller, plants of two other Brassicae species [36]. In our study, all plants were of the same age and plant size did not significantly influence the propensity of females to lay eggs across the two preference experiments. Trichome density, a morphological defensive trait, can also influence female preference and is observed to vary with elevation, though not always in a consistent direction [30]. In the close relative *Arabidopsis lyrata*, the proportion of trichome producing plants declines with increasing elevation consistent with a decline in herbivore pressure [71], and *A. lyrata* genotypes with higher trichome densities showed increased resistance to *P. xylostella*, and accumulated lower numbers of eggs in a field experiment [35]. Similarly, ‘hairy’ genotypes of *A. halleri* ssp. *gemmifera* hosted reduced densities of larvae of *Pieris napi* and a sawfly (*Athalia infumata*) than ‘glabrous’ plants without trichomes [34]. By contrast, in *Arabis alpina*, which shows on average much higher numbers of leaf trichomes than *A. halleri* ssp. *halleri*, trichome density varied in a non-linear manner with elevation [6].

Physiological factors other than those directly involved in defense could also drive the reduced preference for high-elevation populations. For example, flavonoids have been associated with cold-stress tolerance and protection from UV radiation [72], which could be constitutively elevated in populations from high elevations e.g., [73] and therefore impact herbivore oviposition preference [74]. Flavonoids are also one of the few groups of compounds that are consistently produced at higher levels with increasing elevation [3]. Although additional data is needed to disentangle the causal effects of the above factors, our results provide initial insights into how variation in defense investment along elevation gradients can affect plant interactions with their specialist herbivores.

The difference in *P. brassicae* oviposition preference for populations from low and high elevations, but absence of any differences in offspring performance in this study suggests that the oviposition cues that females are using are not predictive of larval survival and growth. Similar performance of larvae on the two high- and two low-elevation populations further suggests that the larvae are unaffected by differences in glucosinolate profiles among *A. halleri* populations from different elevations. Consistent with this observation, *Pieris rapae* larvae performed equally well on *B. oleracea* and *A. thaliana* differing in glucosinolate composition [38,46,57]. By contrast, *P. rapae* and *P. brassicae* larvae feeding on populations of *B. oleracea* with high proportions of indole glucosinolates showed poorer development and performance [64,75]. As we only assayed larval mass gain after a 7-day period, it is possible that our study missed the negative effects of glucosinolate profiles observed for later stages of larval development in those studies. Furthermore, it should also be noted that we tested larval performance on plants that varied most in female preference, coincidentally being also the lowest and highest elevation populations, but not necessarily those with the greatest difference in mean indole glucosinolate concentrations (see values in Table S3). Other work on the defense response of *A. halleri* to herbivory has shown that the hyper-accumulation of heavy metals is more important for this species than glucosinolate induction in defending against invertebrate herbivores [58,76]. However, the relevance of heavy metals to defense of *A. halleri* in our study populations is questionable, as our field sites—some of which are maintained for generating winter cattle feed—are unlikely to be contaminated by heavy metals.

This study is among only a few to date to test for and find variation in herbivore preference for plants originating from different elevations. One previous study assessed the preference of generalist snails for pairs of related species from different elevations, yet found no consistent preference for either montane or low-elevation species in the three pairs tested [31], while another showed that female preference for *Solanum* populations from different elevations was associated with differences in the densities of two types of trichomes [30]. Finally, a study of plant indirect defenses showed that ants preferred the higher volatile emission by high-elevation genotypes of *Vicia sepum* [21]. While specialist herbivores did not vary in performance on plants from different elevations in the current study, female *P. brassicae* clearly showed reduced oviposition preference for plants from high-elevation populations. This reduced preference for high-elevation populations may be linked to the increased investment in indole glucosinolates or to variation in other physiological or morphological traits. While further work is needed to disentangle these different explanatory factors, the current results indicate that population-level variation in traits relevant for adaptation to environments at different elevations can have contrasting effects on insect preference and performance. It is therefore important to consider the full herbivore life cycle for understanding how within species adaptive variation in plant defenses can impact interactions with herbivores. More generally, our results provide insight into the potential consequences of genetic variation in plant defenses along elevation gradients for the herbivore communities with which they interact. Future work should aim to confirm whether the observed defense variation might influence generalist herbivores in the lab and the more complex herbivore communities observed under field conditions.

## 4. Materials and Methods

### 4.1. Establishing Parental Plant Stock Population in the Greenhouse

We sampled and surveyed 13 *A. halleri* populations ranging from 305–2307 m in elevation (see Table S1). Clonal material was collected from field populations in the summer of 2012 (with some populations supplemented with additional collections in 2014 and 2016, see Table S1), by digging up rosettes with intact roots at intervals of 2–5 m (depending on the population size) to minimize sampling of related plants. These parental stock plants were maintained in one greenhouse compartment at the ETH Zürich Research Station for Plant Sciences (Lindau, Effretikon, Switzerland) under controlled temperature conditions (23 °C day, 18 °C night) in clay pots filled with a custom soil mix (consisting of 40 L Substrate 214; 10 L Garden soil, 10 L small expanded clay balls and 5 L perlite, all from Ricoter (Aarberg, Switzerland)). Original parental plants were cloned to produce duplicates of each parental genotype for long-term cultivation. Plants were re-potted at regular intervals and watered three times per week. For experimental work, parental stock plants maintained in a greenhouse for at least one year were cloned and used. Clones were established using cuttings of individual clonal rosettes with intact roots, where possible, and a rooting powder (Neudofix Wurzelaktivator, Emmerthal, Germany) was used to promote root growth and clone establishment. They were then grown at 12 h day (15 Klux): 12 h night (23 °C and 17 °C, 50% and 60% humidity) in a growth chamber.

### 4.2. Assessing Variation in Herbivore Damage across Field Populations

Twelve *A. halleri* populations ranging from 305 m to 2307 m above sea level were visited in 2016 (Table S1). One population, Aha10, could not be surveyed in this year. Populations were visited at an early season time point when plants were expected to be flowering, but the exact survey date varied with elevation, ranging from 28th April 2016 (lowest elevation population) to 5th July 2016 (highest elevation population). A transect was conducted through each population and an 18 cm × 18 cm quadrant placed on the nearest patch of *A. halleri.* Due to the clonal growth of this species, it can be difficult to see individual plants, therefore a minimum distance of 2 m was maintained between patches. We estimated the % area of the quadrant covered by *A. halleri* and recorded the status of plants in the quadrant as either bolting, bolting with developing buds, flowering or fruiting. For each surveyed patch the number of leaves was estimated and the percentage of damaged leaves allocated to one of the six categories described in Table 1. Broad % damage categories were used because of the large numbers of small leaves making precise estimates of leaf area removed too time-consuming to assay sufficient numbers of patches per population (Table 1). Additionally, the presence of different types of leaf damage: leaf holes and damaged leaf edges, was recorded for each patch (see photos in Figure 2).

The effect of elevation on variation in plant damage among populations was therefore analyzed using two response variables: (1) a damage score per patch based on % leaves damaged, and (2) the presence or absence of a damage type (edge damage or holes) on a patch. We used linear regression to test for relationships between leaf damage score per patch and population elevation (with a fixed covariate of patch phenological status). To estimate the amount of variation in leaf damage score associated with population differences, a general linear model with a fixed effect of population was used. The presence or absence of different damage types was analyzed using a generalized linear model with binomial error with fixed effects of either elevation or population, as described above. The significance of population or elevation in these models was tested by comparing a model with the variable to a model with the variable removed (null model) and using F-ratio tests to assess the significance of the change in model explanatory power. All analyses were conducted in the R statistical environment [77].

### 4.3. Assessing the Relationship between Constitutive Glucosinolates in the Greenhouse and Elevation

We sampled three genotypes (or two genotypes for Aha03) from each of eleven populations maintained in the greenhouse. For all populations, except Aha31 and Aha03, we used plants sampled from the field in 2012. Plants from population Aha31 and Aha03 were only sampled from the field in 2014/2016 and 2016 respectively. Glucosinolates were extracted following a published HPLC protocol [78], with several minor modifications described here. Leaves were freeze-dried and ground to a fine powder in a Geno/Grinder 2010 (SPEX sample prep, Metuchen, NJ, USA) for 1 min at 1500 rpm with three 0.3 mm steel grinding balls. After adding 1 mL 70% methanol the extraction samples were heated to 85 °C for 15 min to denature the myrosinase enzyme. Sinigrin reference standards were used to interpolate glucosinolate concentrations based on response factors described in Grosser and van Dam (2017) [78]. Columns were prepared using DEAE (Diethylaminoethyl) Sephadex A25 column material (Sigma-Aldrich, St. Louis, MO, USA). Following elution of samples incubated overnight with sulfatase, samples were dried down on a Savant Speed Vac Concentrator SPP1010 (Thermo Scientific, Reinach, Switzerland) and re-suspended in 150 µL ultrapure MilliQ water (Merck, Darmstadt, Germany). Samples were run on an Agilent 6550 iFunnel Q-TOF LC/MS equipped with an Eclipse XDB-C18 column (4.6 mm × 150 mm, 5 µm, 80 Å) using a water (with 5 mM ammonium formate): acetonitrile gradient. The mobile phase conditions are described by Grosser and van Dam (2017) and consisted of 98% water for 2 min, then a gradient to 65% water over 35 min, followed by a rapid gradient to 2% water over 8 min. The indole glucosinolate, glucobrassicin, was identified through comparison to a pure laboratory standard (Phytoplan Diehm and Neuberger GmbH, Heidelberg, Germany), which was processed separately alongside the samples to produce desulfo-glucobrassicin. As standards were not available for other glucosinolates, desulfo-gluosinolates were identified based on their fragmentation pattern due to the loss of a hexose-derivative from a parent aglycone, demonstrated by a mass shift of 162 amu, and then through formula matches identified using Agilent MassHunter qualitative software. UV spectra of desulfo-glucosinolates were examined to confirm whether the glucosinolate represented either the aliphatic or indole class. We additionally used retention times and the eluotropic logical series of methylsulfinyl- and methylthio-glucosinolates to provisionally identify longer chain aliphatic glucosinolates. As standards were not available, we were unable to distinguish the isomers 4-methoxyglucobrassicin and *N*-methoxyglucobrassicin, but assumed the presence of 4-methoxyglucobrassicin, as it was previously identified in *A. halleri* by [75]. No other indole glucosinolates were detected in our samples, and specifically we did not detect 4-hydroxyglucobrassicin, an intermediate in the biosynthesis of 4-methoxyglucobrassicin from glucobrassicin. We quantified amounts of two indole and seven aliphatic glucosinolates that have previously been identified in *A. halleri* [58,76] (see Table S2). The integration of the 229 nm UV spectrum was used for quantification of compounds based on a comparison to a sinigrin concentration curve and published response factors (as described in [78]). Amounts of glucosinolates were then converted to µmol per gram fresh tissue weight (FW).

Linear regression was used to test for a significant effect of population elevation on total glucosinolates, and total aliphatic or indole glucosinolates separately. Plant size was included as a fixed effect in theses analyses to control for differences in plant growth rates under greenhouse conditions affecting glucosinolate production. Finally, to test whether investment in the two glucosinolate classes was correlated, we also used linear regression to test for a relationship between total aliphatic and total indole glucosinolates.

We then explored whether the observed variation in glucosinolate investment in the greenhouse could be explained by variation in herbivore pressure in the field. For this analysis population mean values of glucosinolates and damage to plant patches in the field were used. We used linear regression to test for an effect of mean field damage score on greenhouse estimates of total aliphatic or total indole glucosinolates. These analyses were repeated using the proportion of patches with leaf edges damaged per population, or the proportion of patches with holes per population, as the explanatory factor. For all the above analyses, an F-ratio test comparing the model with and without the factor of interest was used to test the significance of each explanatory factor and log transformations were used to improve model fit where deviations in model residuals were observed.

### 4.4. Screening Populations of A. halleri for Variation in Butterfly Oviposition Preference

Clones of eight populations were used in the following experiments. An average of four clones (ranging from 2 to 7 depending on the size of the parental plant) were produced from each of 4–7 parental genotypes per population. Fertilizer was added to promote clone growth and *Bacillus thuringiensis* was added early in the growth period to control fungus gnats. Senescing leaves were initially removed to promote further leaf growth, but this was stopped 1 month prior to the experiment.

#### 4.4.1. Preference Experiment 1: Assessing Population-Level Effects on Preference

For each of the eight populations, four genotypes with 2–3 healthy clones were selected (10–12 individual plants per population; Table S1). Due to space limitations, these experiments were conducted in three rounds with, where possible, a clone of each genotype represented in each experimental round. For each round, four plants were randomly allocated to each of eight net cages, split between two well-lit, temperature-controlled rooms. Female *Pieris brassicae* that were maintained for multiple generations in the lab on *Brassica oleracea* were used in the experiment. Initially, one gravid female *P. brassicae* was added to each cage and egg-laying behavior monitored over a 20-min period. If this butterfly did not lay eggs, it was replaced by another female and the 20-min monitoring restarted. We recorded the number of eggs laid after 20 min. Following this short observation period, we put two females in each cage and left them until mid-morning the following day (approximately 20 h) when the cumulative total number of eggs laid on the plants after this period were counted. Some plants showed evidence of leaf senescence, so plant health was estimated using a categorical scale based on proportion of leaves showing minor discoloring (red, yellow or brown patches): (1) <5% leaves discolored, (2) ≤25%, (3) ≤50%). Plants with >50% discolored leaves were not used in experiments. Additionally, average rosette width was calculated from two perpendicular width measurements, and maximum rosette height from the soil surface was recorded.

As low numbers of eggs were laid in the initial 20-min period, we focused our analyses on the total numbers of eggs laid after 20 h in the cage. We used a binomial Generalised Linear Model (GLM) to test for an effect of plant population on the propensity to lay eggs (a presence or absence binomial response variable). The egg count data showed high overdispersion when modelled with a Poisson error family. Given the dataset also potentially contained false zeros (females that that were unmated and could not lay eggs), we used a zero-inflated negative binomial model to test the effect of population (in the ‘pscl’ package in the R statistical software, [79]). We assumed a constant probability of false zeros across populations and retained a fixed factor of experimental round to control for variance among experimental repetitions. We also tested for a population effect on the number of eggs laid after excluding plants on which no eggs were laid. For this, a negative binomial model without zero-inflation was used. A separate zero-inflated negative binomial model analysis tested for significant effects of rosette width, plant height and plant health score on variation in the number of eggs laid after 20 h, with experimental round again included in the model (model formula: number eggs ~ rosette width + plant height + plant health + experimental round). One outlier point from Aha21 (with 417 eggs) is not shown on the plot to aid visualization of other points. The effect of population (first model) and all other explanatory factors (second model) was tested by removing non-significant factors and then comparing models with or without the factor of interest using likelihood ratio tests. Model residuals were inspected and the effect of any outlier points on model results checked.

#### 4.4.2. Preference Experiment 2: Assessing Elevation Effects on *P. brassicae* Preference

We then used plants from four populations (Aha18, Aha31, AhaN4, Aha19) to repeat the preference assay, as well as to test whether female preference was related to larval performance. Specifically, we used three to six genotypes per population, and the number of clones per genotype ranged from one to four, with a total of nine clones per population. The same host choice and egg laying measurements as described above were recorded after a 20 h overnight period. A binomial model was used to test for the effect of population and rosette area on the presence or absence of eggs on test plants after 20 h. A zero-inflated negative binomial model was again used to model the effect of population and rosette area on the number of eggs.

Immediately following this preference assay, all plants were infested with eggs and larvae were allowed to hatch. Excess larvae were removed as soon as eggs hatched to leave ten L1 larvae per plant. These were allowed to feed on the plants for 7 days, and their individual mass was measured at the end of the 3rd and 7th day. By the 7th day, the larvae had consumed most of the plant leaves, so we were unable to take leaf samples for chemical defense analysis. Variation in individual larval mass was modelled with a linear mixed effects model (R package, lme4; [80]) using population as a fixed factor and individual plant as a random factor. Estimates of the variance explained by the fixed and random terms were calculated using the package “piecewiseSEM” [81].

## Figures and Tables

**Table 1:** Damage scores used for categorizing extent of damage to different plant patches, based on the percentage of leaves showing any signs of damage and placed in to one of six broad categories.

% Leaves Damaged	Score
<1%	0
1–5%	1
5–10%	2
10–25%	3
25–50%	4
>50%	5

## Supplementary Materials

Supplementary materials can be found at http://www.mdpi.com/1422-0067/20/1/174/s1, Figure S1: Regression of total aliphatic glucosinolates against elevation controlling for variation in plant size, Figure S2: Regressions of amounts of aliphatic and indole glucosinolates against each other and with respect to population-level mean field damage, Table S1: Overview of locations of *Arabidopsis halleri* study sites in the Alps, as well as numbers of genotypes and plants used in different parts of the study, Table S2: Glucosinolates identified in *A. halleri* in our study, with the abbreviations used throughout the manuscript, and the retention times on our LC/MS system, Table S3: Mean amounts of total, aliphatic and indole glucosinolates for each population screened in this study, Table S4: Overview of glucosinolate investment, field damage and *Pieris brassicae* preference for four populations (two high-elevation, two low-elevation) used in the second preference assay.
