# Supplementary material for: Divergence in Glucosinolate Profiles between High- and Low-Elevation Populations of Arabidopsis halleri Correspond to Variation in Field Herbivory and Herbivore Behavioral Preferences
Source: Int J Mol Sci. 2019 Jan 5;20(1):174. doi: 10.3390/ijms20010174 (PMC6337533; doi:10.3390/ijms20010174)
Supplement: Supplementary file 1 [file ijms-20-00174-s001.pdf]

Divergence in Glucosinolate Profiles between High- and Low-Elevation Populations of *Arabidopsis halleri* Correspond to Variation in Field Herbivory and Herbivore Behavioral Preferences

James Buckley, Foteini G. Pashalidou, Martin C. Fischer, Alex Widmer, Mark C. Mescher, Consuelo M. De Moraes*

SUPPLEMENTARY INFORMATION

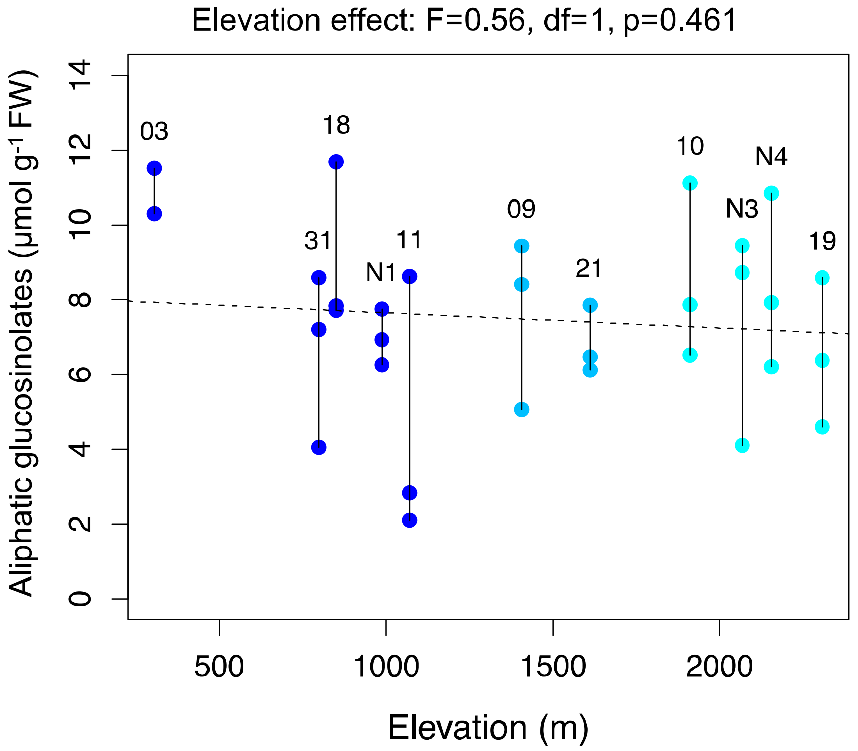

Figure S1. Regression of total aliphatic glucosinolates (in micromoles per gram of fresh tissue, μmol g^{-1} FW) against elevation controlling for variation in plant size (with samples from the same population joined by black vertical lines). Samples came from plants growing under a common greenhouse environment. Elevation had no significant effect on aliphatic glucosinolate concentrations. Points are coloured by elevation class.

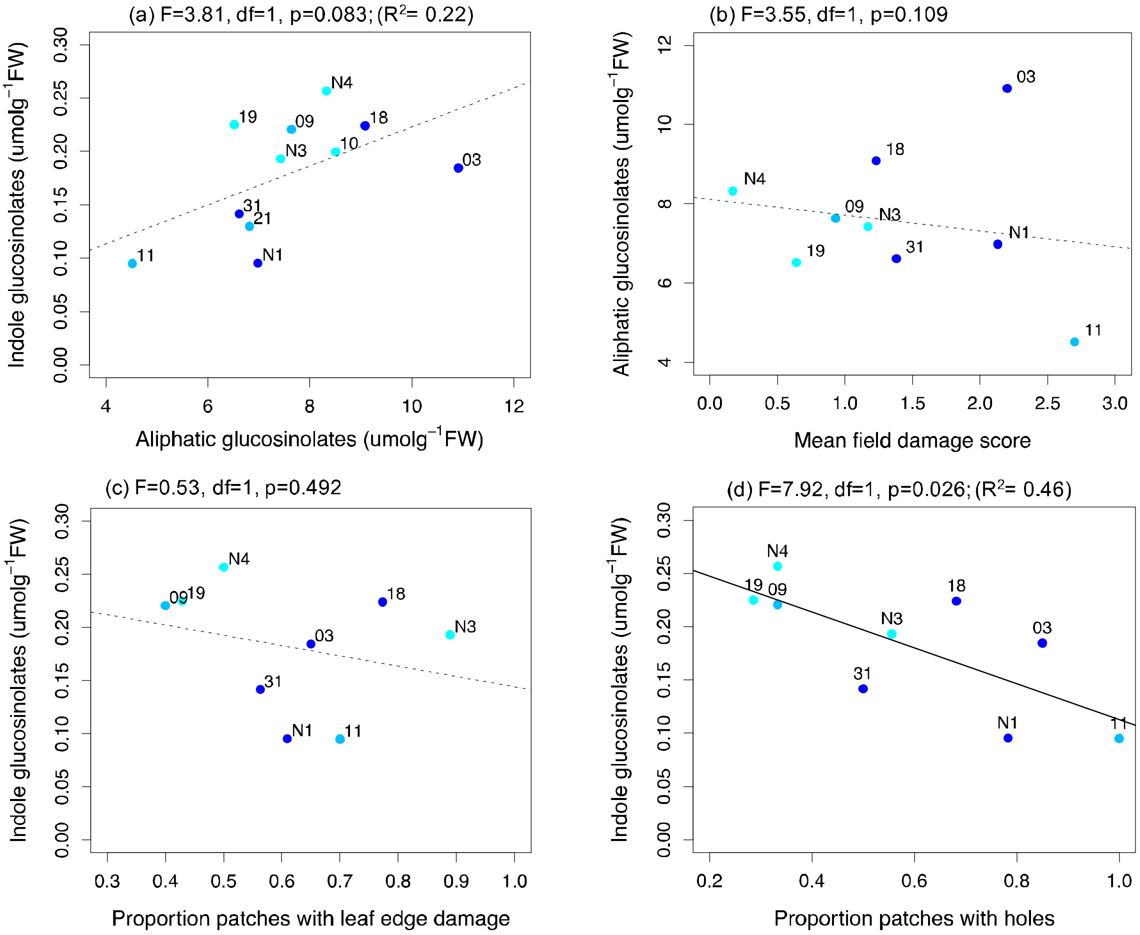

Figure S2. Regressions of amounts of aliphatic and indole glucosinolates (in micromoles per gram of fresh tissue, µmol g-1 FW) against each other and with respect to population-level mean field damage. (**a**) total indole glucosinolates against total aliphatic glucosinolates. (**b**) total aliphatic glucosinolates against mean damage score per population in the field surveys in 2016. (**c**) total indole glucosinolates against the proportion of patches in a population with leaf edge damage. (**d**) total indole glucosinolates against the proportion of patches in a population with hole damage. The significance of the effect and adjusted R-squared is given. Points are coloured by elevation class. A dashed line indicates a non-significant regression line, a solid line indicates a significant line.

Table S1. Overview of locations of study sites in the Alps, including a brief description of the habitat in which *Arabidopsis halleri* was found, as well as the dates on which damage surveys were conducted in the field and the number of genotypes (and total number of individual plants) used for preference assays and screened for constitutive variation in glucosinolates (GSLs). Elevation is given in metres above sea level (m asl).

							Number genotypes	
**Site name**	**Swiss Canton**	**Coordinates (decimal degrees, °N,°E)**	**Elevation (m asl)**	**Habitat description**	**Year parent collected**	**Field damage survey date**	**Preference assay (total number)**	**Screening GSLs**
Aha03	Ticino	46.17733, 8.70761	305	Roadside verge, forest edge	2016	29th April	-	2
Aha31	Graubünden	46.33682, 9.52171	798	Grass meadow (for cutting)	2014, 2016	29th April	4 (12)	3
Aha01	Ticino	46.16439, 9.07098	804	Grass meadow around building	2016	28th April	-	-
Aha18	Ticino	46.41593, 8.82605	850	Grass meadow (for cutting) and forest glade	2012	28th April	4 (12)	3
Aha02	Ticino	46.16814, 9.09173	944	Grass meadow (for cutting)	2016	28th April	-	-
AhaN1	Graubünden	46.27532, 10.10072	987	Next to path, grass meadow (for cutting)	2012	30th April	4 (12)	3
Aha11	Graubünden	46.27767, 10.10619	1070	Grass meadow around building	2012	30th April	-	3
Aha09	Graubünden	46.36925, 9.65868	1406	Grass meadow (for cutting)	2012	29th April	4 (12)	3
Aha21	Graubünden	46.36682, 9.63081	1611	Grass meadow around building	2012	Not done	4 (11)	3
Aha10	Graubünden	46.45919, 9.88106	1910	Grass meadow around building	2012	Not done	4 (11)	3
AhaN3	Graubünden	46.49926, 9.82731	2067	Vegetated areas around buildings	2012	4th July	-	3
AhaN4	Italy	46.49115, 10.22226	2155	Grass meadow	2012, 2016	5th July	4 (11)	3
Aha19	Graubünden	46.41125, 10.02253	2307	Grass meadow (grazed)	2012, 2016	5th July	4 (10)	3

Table S2. Glucosinolates identified in A. halleri in our study, with the abbreviations used throughout the manuscript, together with the retention times and m/z values ([M-H]- ion) of desulfo-glucosinolates (desulfo-GSL) on our LC/MS system. Sinigrin monohydrate was used as a standard for quantification of other glucosinolates and is therefore highlighted in bold (abbreviation 'PREN'). All the aliphatic glucosinolates listed derive from the amino acid methionine, and the listed indole glucosinolates derive from the amino acid tryptophan. All glucosinolates were provisionally identified by MS formula matches, UV spectra and retention times (eluotropic series), but the identity of glucobrassicin (GB) was also confirmed through comparison to a laboratory standard.

Abbreviation	Full name (+GSL suffix)	Glucosinolate class	Other names	Retention time	m/z ([M-H]-)	Formula (desulfo-GSL)
**PREN**	**2-propenyl**	**aliphatic**	**Sinigrin**	**4.9**	**277.07**	$\mathbf{C_{10}H_{12}NO_6S}$
5MSP	5-(methylsulfinyl)pentyl	aliphatic	Glucoalyssin	6.63	370.09	$C_{13}H_{25}NO_7S_2$
6MSH	6-(methylsulfinyl)hexyl	aliphatic	Glucohesperin	9.35	384.12	$C_{14}H_{27}NO_7S_2$
7MSH	7-(methylsulfinyl)heptyl	aliphatic	Glucoibarin	12.6	398.14	$C_{15}H_{29}NO_7S_2$
GB	3-indolylmethyl	indole	Glucobrassicin	14.35	367.09	$C_{16}H_{20}N_2O_6S$
8MSO	8-(methylsulfinyl)octyl	aliphatic	Glucohirsutin	16.5	412.15	$C_{16}H_{31}NO_7S_2$
4MeOGB	4-methoxy-3-indolylmethyl	indole	4-Methoxyglucobrassicin	17.35	397.11	$C_{17}H_{22}N_2O_7S$
6MTH	6-(methylthio)hexyl	aliphatic	Glucolesquerellin	23.05	368.12	$C_{14}H_{27}NO_6S_2$
7MTH	7-(methylthio)heptyl	aliphatic	NA	28.05	382.137	$C_{15}H_{29}NO_6S_3$
8MTO	8-(methylthio)octyl	aliphatic	NA	32.85	396.15	$C_{16}H_{31}NO_6S_2$

Table S3. Mean amounts of total, aliphatic and indole glucosinolates for each population screened in this study (in micromoles per gram of fresh tissue, μmol g^{-1} FW). The proportion of indole glucosinolates relative to the total glucosinolates is given and populations are ordered by increasing elevation. The values in each column are shaded red to indicate populations with relatively high amounts (darker reds) and low amounts (white/lighter reds) of the respective glucosinolate class.

		Mean amounts in μmol g^{-1} FW			
**Population**	N	Total glucosinolates	Total aliphatics	Total indoles	% indoles
Aha03	2	11.10	10.91	0.18	1.7
Aha31	3	6.76	6.62	0.14	2.1
Aha18	3	9.31	9.08	0.22	2.4
AhaN1	3	7.08	6.98	0.10	1.3
Aha11	3	4.61	4.52	0.09	2.1
Aha09	3	7.86	7.64	0.22	2.8
Aha21	3	6.95	6.82	0.13	1.9
Aha10	3	8.71	8.51	0.20	2.3
AhaN3	3	7.62	7.43	0.19	2.5
Aha19	3	6.75	6.52	0.22	3.3
AhaN4	3	8.58	8.33	0.26	3.0

Table S4. Overview of glucosinolate investment, field damage and *Pieris brassicae* preference for the four populations (two high-elevation, two low-elevation) used in the second preference experiment. The mean number of eggs laid after 20 h are given for the four populations used in the first and second preference experiment, as well as the mean amounts of total, aliphatic and indole glucosinolates (in micromoles per gram of fresh tissue, μmol g^{-1} FW) estimated under greenhouse conditions. The percentage of indole glucosinolates relative to the total glucosinolates is given. The last column provides mean damage score per plant surveyed in the field for the four populations. .

		Preference experiment 2		Preference experiment 1		Mean μmol g^{-1} FW				
**Population**	**Elevation class**	**Mean eggs laid**	**SE**	**Mean eggs laid**	**SE**	**Total glucosinolates**	**Total aliphatics**	**Total indoles**	**% indoles of total**	**Mean damage score (field)**
Aha31	low	18.0	7.9	23.3	11.5	6.76	6.62	0.14	2.1	1.38
Aha18	low	22.8	11.6	27.2	8.2	9.31	9.08	0.22	2.4	1.23
AhaN4	high	7.3	3.4	11.6	6.2	8.58	8.33	0.26	3.0	0.17
Aha19	high	0.7	0.4	4.9	2.4	6.75	6.52	0.22	3.3	0.64
